# Impact of an end-of-fourth-year emergency medicine bootcamp

**DOI:** 10.1186/s12245-021-00371-8

**Published:** 2021-09-03

**Authors:** Jason J. Lewis, Anne V. Grossestreuer, Edward A. Ullman

**Affiliations:** Department of Emergency Medicine, Beth Israel Deaconess Medical Center, One Deaconess Road, Rosenberg Building 2, Boston, MA 02215 USA

**Keywords:** Bootcamp, Boot camp, Undergraduate medical education, Emergency medicine education, Transition course, Capstone course

## Abstract

**Background:**

The final months of the fourth-year of medical school are variable in educational and clinical experience, and the effect on clinical knowledge and preparedness for residency is unclear. Specialty-specific “bootcamps” are a growing trend in medical education aimed at increasing clinical knowledge, procedural skills, and confidence prior to the start of residency.

**Methods:**

We developed a 4-week Emergency Medicine (EM) bootcamp offered during the final month of medical school. At the conclusion of the course, participants evaluated its impact. EM residency-matched participants and non-participants were asked to self-evaluate their clinical knowledge, procedural skills and confidence 1 month into the start of residency. Program directors were surveyed to assess participants and non-participants across the same domains. A Fisher’s exact test was performed to test whether responses between participants and non-participants were statistically different.

**Results:**

From 2015 to 2018, 22 students participated in the bootcamp. The majority reported improved confidence, competence, and procedural skills upon completion of the course. Self-assessed confidence was significantly higher in EM-matched participants 1 month into residency compared to EM-matched non-participants (*p* = 0.009). Self-assessed clinical knowledge and procedural skill competency was higher in participants than non-participants but did not reach statistical significance. Program directors rated EM-matched participants higher in all domains but this difference was also not statistically significant.

**Conclusions:**

Participation in an EM bootcamp increases self-confidence at the start of residency among EM-matched residents. EM bootcamps and other specialty-specific courses at the end of medical school may ease the transition from student to clinician and may improve clinical knowledge and procedural skills.

**Supplementary Information:**

The online version contains supplementary material available at 10.1186/s12245-021-00371-8.

## Introduction

The transition from medical student to resident physician is a formative and difficult process. Currently, the final months of the fourth-year in medical school are variable in both educational and clinical experience. The effect on clinical knowledge and procedural competence is unclear. Specialty-specific education prior to the start of residency is essential to increase clinical knowledge, procedural skills, and confidence at the start of internship [1–6]. Intensive, specialty-specific “bootcamps” at the end of medical school are a growing trend in medical education designed to achieve these goals [2, 5]. Transition or “capstone” courses can help prepare students for the professional and personal challenges of progressing from medical student to resident, but these are often not specialty-specific [7–9].

Emergency medicine (EM) is a unique residency requiring immediate proficiency in a wide variety of clinical procedures and a broad clinical knowledge in order to provide safe and effective patient care in the emergency department (ED). While those matching in EM have typically completed two to four EM clerkships during their undergraduate medical education, students typically participate in their clerkships during the beginning months of their fourth-year. The variability of clinical experience post-interview season and end-of-fourth-year electives can have a significant impact on new interns’ clinical knowledge, clinical practice, and confidence at the start of residency. This is supported by previous work that has shown that often there is a deficiency in procedural knowledge and confidence of medical students seeking a career in EM [2].

In response to this variability, we created a 4-week intensive EM bootcamp comprised of case-based lectures, high-fidelity simulation, and procedural skill sessions critical to success in EM residency. While previous EM bootcamps have been offered to medical students in a 1-day procedurally focused session or to EM interns in a 2-day or 5-day model, none describe a dedicated 4-week elective course in EM designed for graduating fourth-year medical students matching in EM [1–3]. A review of the literature in MedEdPORTAL searching for “boot camp,” “bootcamp,” “short immersive course,” “pre-residency,” “pre-internship,” “pre internship,” and “pre residency” education returned 16 pertinent results [10–25], only one of which described an EM-specific curriculum [18]. This was a simulation-based curriculum offered to interns during orientation, which showed improvement in confidence and knowledge; however, this was a dedicated resident-based curriculum [18]. The goal of this study was to determine the impact of participation in a 4-week intensive EM-specific bootcamp offered during the final month of medical school on clinical knowledge, procedural skill competency, and intern confidence at the start of residency.

## Methods

### Study design

This was a prospective survey study conducted at an urban, tertiary care academic medical center in Boston, MA, affiliated with Harvard Medical School. We developed and implemented a 4-week EM bootcamp at the affiliated medical school based on a needs assessment of previous graduates matching in EM, including one of the authors, as well as input from core medical education faculty. The course focuses on clinical knowledge and procedural skills that were identified as lacking at the beginning of residency. The curriculum consists of case-based clinical lectures and generalizable residency topics, resident led didactics on day-to-day activities, procedural skills sessions, four high-fidelity simulation sessions, and an intensive ultrasound curriculum (see Additional file 1). The course runs Monday through Friday for 4 weeks with students also participating in weekly residency program didactics (see Additional file 2). All bootcamp participants were surveyed at the end of the course regarding the immediate impact as part of the anonymous post-course evaluation. All EM-matched participants and non-participants as well as their program directors (PDs) were subsequently surveyed 1 month into the start of residency to assess further effect. This study was reviewed and determined exempt by our institution’s Institutional Review Board.

### Study setting and population

From 2015 to 2017, all graduating medical students matching in EM from our affiliated medical school were offered the opportunity to participate in the EM bootcamp. Starting in 2018, all graduating medical students were offered the opportunity to participate regardless of matched specialty. All bootcamp participants completed a post-course evaluation. EM-matched students (both participants and non-participants) were surveyed 1 month into the start of residency.

### Study protocol and outcome measures

At the end of the bootcamp, all participants evaluated the impact of the bootcamp on their confidence, clinical knowledge, and procedural competencies on a 1–4 Likert scale as part of the anonymous post-course evaluation (see Additional file 3). All EM-matched graduates and their PDs were anonymously surveyed 1 month into the start of intern year to assess their clinical knowledge, procedural skills, and confidence on a 1–5 Likert scale (see Additional file 4 and Additional file 5).

### Statistical analysis

Results of the PD and self-assessment surveys were analyzed using a Fisher’s exact test to compare EM-matched bootcamp participants versus EM-matched non-participants across the three domains. Analysis was performed using Stata 14.2 (College Station, TX) and a *p* value < 0.05 was considered statistically significant.

## Results

From 2015 through 2018, 39 students from our affiliated medical school matched in EM and 18 participated in the boot camp (Table 1). In 2018, four non-EM-matched students participated: two matching in radiology, one in medicine and one in pediatrics for a total of 22 participants from 2015 to 2018. All 22 of the participating students completed the anonymous post-course evaluation. Over 90% of students reported a moderate to significant impact on confidence, competence and procedural skills and over 95% would recommend the course without reservation to the next year’s class (Table 2).

**Table 1:** EM-matched graduates from 2015 to 2018 (*n* = 39)

Demographics	Participants (*n* = 18)	Non-participants (*n* = 21)	*p* value
Male	12 (66.7)	11 (52.4)	0.516
Year			
2015	7 (38.9)	7 (33.3)	0.172
2016	6 (33.3)	3 (14.3)	
2017	3 (16.7)	10 (47.6)	
2018	2 (11.1)	1 (4.8)

**Table 2:** Post-course participant evaluation (*n* = 22)

Question	Response
How has this course increased your confidence?	
Significantly	8 (36.4)
Moderately	12 (54.5)
Slightly	2 (9.1)
Not at all	0 (0)
How has this course increased your competence?	
Significantly	8 (36.4)
Moderately	12 (54.5)
Slightly	2 (9.1)
Not at all	0 (0)
How has this course impacted your procedural skills?	
Significantly	11 (50.0)
Moderately	9 (40.9)
Slightly	2 (9.1)
Not at all	0 (0)
Would you recommend this course to next year’s graduating class?	
Absolutely	21 (95.5)
Probably yes	1 (4.5)
Probably no	0 (0)
No	0 (0)
End of course comments:	“Thank you so much for putting this course together. It was a great primer for starting intern year and makes me feel a lot more confident about starting. More importantly, it helped me realize how much I don’t know, but made me feel comfortable that its ok, and I will learn.” “Thoroughly enjoyed the course.” “Great course!” “The overall schedule struck the right balance for students who are passionate about EM but in the homestretch.” “I loved almost everything!!! Awesome lectures that were to the point and appropriately challenging for our level but focused on key ‘intern level take home points’…And loved the skills and sim sessions. Awesome awesome awesome course.” “Really grateful for this course and feeling re-inspired and reinvigorated about EM.” “Informal exposure to residents before/after/during sessions they were leading was particularly valuable.” “I really appreciate the effort from the course directors and course faculty to make this an outstanding course.”

Twenty-nine interns (74%) responded to the survey and program directors completed the survey for 29 graduates (74%). Self-assessed confidence was significantly higher in participants versus non-participants (*p* = 0.009) with 45.5% of non-participants reporting below average self-confidence, while only one participant noted below average confidence. Self-assessed clinical knowledge and procedural skills showed improvement but did not reach statistical significance (Table 3). PDs rated more participants than non-participants above average across all domains, but these results also did not reach statistical significance (Table 4). No participants were noted to be below average, while 2 out of 15 (13%) assessed non-participants were rated moderately to significantly below average in each domain (Table 4).

**Table 3:** EM intern self-assessment of clinical knowledge, procedural skills, and confidence for bootcamp participants and non-participants

Question and response	Participants (*n* = 18)	Non-participants (*n* = 11)	*p* value
How do you feel your clinical knowledge compares to your co-interns?			
Significantly below	0 (0)	0 (0)	0.461
Moderately below	0 (0)	0 (0)	
Average	12 (66.7)	10 (90.9)	
Moderately above	5 (27.8)	1 (9.1)	
Significantly above	1 (5.6)	0 (0)	
How do you feel your procedural skills compare to your co-interns?			
Significantly below	0 (0)	1 (9.1)	0.229
Moderately below	1 (5.6)	3 (27.3)	
Average	8 (44.4)	4 (36.4)	
Moderately above	6 (33.3)	3 (27.3)	
Significantly above	3 (16.7)	0 (0)	
How confident were you at the start of internship?			
No confidence	0 (0)	3 (27.3)	0.009
Mild lack of confidence	1 (5.6)	2 (18.2)	
Average	7 (38.9)	5 (45.5)	
Mildly confident	10 (55.6)	1 (9.1)	
Very confident	0 (0)	0 (0)

**Table 4:** Program director evaluation of EM bootcamp participants’ and non-participants’ clinical knowledge, procedural skills, and confidence

Question and response	Participants (*n* = 14)	Non-participants (*n* = 15)	*p* value
How does this intern’s clinical knowledge compare to your other interns?			
Significantly below	0 (0)	0 (0)	0.473
Moderately below	0 (0)	2 (13.3)	
Average	4 (28.6)	6 (40.0)	
Moderately above	9 (64.3)	6 (40.0)	
Significantly above	1 (7.1)	1 (6.7)	
How does this intern’s procedural skills compare with your other interns?			
Significantly below	0 (0)	0 (0)	0.651
Moderately below	0 (0)	2 (13.3)	
Average	10 (71.4)	11 (73.3)	
Moderately above	3 (21.4)	2 (13.3)	
Significantly above	1 (7.1)	0 (0)	
How would you rate this intern’s confidence compared with other interns in your program?			
Significantly below	0 (0)	1 (6.7)	>0.999
Moderately below	0 (0)	1 (6.7)	
Average	9 (64.3)	9 (60.0)	
Moderately above	4 (28.6)	4 (26.7)	
Significantly above	1 (7.1)	0 (0)	

## Discussion

The transition from medical student to resident is difficult. This is further exacerbated by the particular demanding environment of patient care in the ED. This transition can be eased with increased competence and confidence. In this study, participation in a 4-week EM bootcamp at the end of medical school improved resident confidence and showed a promising impact on clinical knowledge and procedural skills.

While there was only a significant difference in self-assessed confidence at the start of residency, there were positive trends in all participants in self-assessed clinical knowledge and procedural skill competence as well as across all three domains on PD assessment as compared to non-participants. This may have been influenced by self-selection and inherent variability of students who chose to participate versus those who did not, with those participating potentially feeling that they lacked EM-specific knowledge and skills. Verbal and written feedback from participants at the completion of the course highlighted the benefits of the course, one of which was a realization of continued knowledge gaps: “More importantly, it helped me realize how much I don’t know, but made me feel comfortable that its ok, and I will learn” (See Table 2). This may have continued to affect their self-assessed knowledge and skills at the start of residency versus those non-participants who were not alerted to any potential knowledge deficiencies. Furthermore, our results may have been impacted by the Dunning-Kruger effect, whereby non-participants with possible lower skill ability overestimate their abilities [26]. Finally, it is possible that there were significant differences in the depth, breadth and length of intern orientation for each residency program, which may have impacted the intern’s perception of their skills, knowledge and confidence.

The use of bootcamps has been shown to improve learners’ clinical skills, knowledge, and confidence, which corroborates our current findings [4]. While other studies have shown that an end-of-medical school EM bootcamp focusing on 1 day of procedural skills and a 4-week surgical bootcamp improved participant confidence, this is the first month-long course to incorporate high impact educational modalities into over 100 h of EM-specific education at the conclusion of medical school [2, 5]. Likewise, other studies have shown increased preparedness and improved confidence after participation in a 5-day EM-specific pre-orientation workshop, a 2-day EM intern bootcamp, and a simulation-based EM intern orientation curriculum, but these courses are site-specific and offered during residency, which may limit the participant’s focus given other more demanding aspects of starting residency [1, 3, 18]. This course was developed based on a needs assessment of the previous graduates matching in EM as well as core EM medical education faculty to specifically target deficiencies in EM-specific undergraduate medical education.

While there are several administrative challenges, primarily in terms of time and personnel, in implementing a new 4-week EM intensive elective course, we feel this curriculum is potentially generalizable and adaptable to all undergraduate medical institutions. There is need for significant support from the academic faculty as there are over 20 h of dedicated attending lectures. We chose educators who already had a proven history of lecturing to EM residents and faculty. Furthermore, the course does require significant simulation time as we utilized over 20 h during the course. It is unclear if other institutions have these resources readily available.

Additionally, since this course was an elective, the number of participants varies from year to year. In 2017, we had less than 25% of students matching in EM participate in the bootcamp. It is unclear what precipitated this to occur and may have been impacted by the need to complete other medical school graduation requirements or that the majority of EM-matched students felt that they were prepared for residency and did not require additional instruction. In 2018, fewer students matched in EM, leading to fewer participants in the course. We found that the course is very adaptable to fluctuating numbers of students and did not require any alterations based on participant number. Once the generalized schedule format, lectures, and simulation cases were developed, there were no changes that needed to be made with varying numbers of students. Given the academic mission of our department and our affiliated medical school’s requirement for 50 h of medical student teaching per year, it is still beneficial to run the course regardless of enrollment numbers. However, we acknowledge that at other institutions with fewer available resources, this may be more difficult. Nevertheless, the general structure of our curriculum with mixed educational approaches of simulation, high-yield clinical didactics, resident-based lectures, and procedural skills sessions can be adapted to any medical school’s needs.

## Limitations

First, this study may have limited generalizability due to its single-center design, relatively small sample size, and the potential for self-selection among students opting to take/not take the bootcamp. However, graduating EM-matched students matched into 23 different EM residency programs, increasing the heterogeneity of the results. Second, the surveys are a subjective measure of intern self-assessment and PD assessment and it is unclear how these results translate to clinical practice. Third, given the self-assessment of skills by new interns, it is also possible that non-participant individuals were impacted by the Dunning-Kruger effect, although this would bias our results toward the null [26]. Furthermore, since we did not obtain information on the length and breadth of intern orientation for each residency program, differences in teaching during those times may have impacted the results. Finally, since PDs were not blinded to the intern’s participation status, they may have been biased on assessing interns based on whether or not they participated in the bootcamp.

## Conclusions

Participation in this unique 4-week intensive end-of-year EM bootcamp significantly increased self-confidence at the start of residency and adds support to the benefits of specialty-specific bootcamps at the end of medical school. There is also possible impact on clinical knowledge and procedural skills. This course may ease the transition from student to clinician and potentially can be adapted to any department or institution’s individual needs to improve medical student pre-residency education.

## Supplementary Information

**Additional file 1:.** Emergency Medicine Bootcamp Curriculum

**Additional file 2:.** Weekly Bootcamp Schedule

**Additional file 3:.** End of Bootcamp Course Evaluation

**Additional file 4:.** Emergency Medicine Bootcamp New Intern Survey

**Additional file 5:.** Emergency Medicine Bootcamp Program Director Survey

## Data Availability

The datasets used and/or analyzed during the current study are available from the corresponding author on reasonable request.

## Abbreviations

EM: Emergency medicine
ED: Emergency department
PD: Program director
