# Supplementary material for: Impact of an end-of-fourth-year emergency medicine bootcamp
Source: Int J Emerg Med. 2021 Sep 3;14:48. doi: 10.1186/s12245-021-00371-8 (PMC8414734; doi:10.1186/s12245-021-00371-8)
Supplement: Supplementary file 1 — Additional file 1:. Emergency Medicine Bootcamp Curriculum [file 12245_2021_371_MOESM1_ESM.docx]

Additional file 1 - Emergency Medicine Bootcamp Curriculum

**Attending Lectures:**

1. **Introduction to Emergency Radiology**

By the end of this session, participants will:

- - 1. Learn approach to common ED performed radiographic studies
    2. Interpret common ED radiographic findings

1. **Urologic Emergencies**

By the end of this session, participants will:

- - 1. Understand the most common urologic presentations in the ED
    2. Know when to consult urology
    3. Know how to perform basic urologic procedures in the ED

1. **Introduction to EM Practice**

By the end of this session, participants will:

- - 1. Start thinking about how to develop systematic practice patterns
    2. Arrive to residency with some basic approaches to patients and chief complaint specific differentials
    3. Start thinking about ways to best build clinical skills and knowledge

1. **Introduction to the ED Eye Exam**

By the end of this session, participants will:

- - 1. Understand the components of the slit lamp exam
    2. Learn how to use the slit lamp and do a full eye exam
    3. Learn how to appropriately document the exam

1. **Hand Emergencies**

By the end of this session, participants will:

- - 1. Understand the intricate anatomy of hand emergencies
    2. Expand their practice of hand procedures in the ED
    3. Know when to call the hand specialist

1. **Environmental Emergencies**

By the end of this session, participants will:

- - 1. Demonstrate understanding of the physiology, diagnosis and treatment of common environmental hazards related to weather (heat, cold, lightning strike)
    2. Demonstrate understanding of the physiology, diagnosis and treatment of common environmental hazards related to physical environments (altitude, submersion injuries)
    3. Demonstrate understanding of the physiology, diagnosis and treatment of common environmental hazards related to animal exposures (snake envenomation, spider bites, marine envenomation)

1. **Cultural Competence**

By the end of this session, participants will:

- - 1. Explain how cultural beliefs influence an individual’s approach to health, illness and treatment
    2. Recognize tendencies toward bias and stereotyping
    3. Discuss racial and ethnic disparities in healthcare

1. **Trauma**

By the end of this session, participants will:

- - 1. Learn how to diagnose injuries in the unstable blunt trauma patient
    2. Learn the initial management and stabilization of patients with blunt trauma
    3. Learn indications for emergency department thoracotomy

1. **Approach to the Acute Abdomen**

By the end of this session, participants will:

- - 1. Recognize high-risk features of the common chief complaint of abdominal pain
    2. Describe the utility of ultrasound, plain radiography, CT, and MRI in working up abdominal pain
    3. Construct a differential diagnosis for nausea and vomiting that includes etiologies beyond GI illnesses

1. **Pediatrics**

By the end of this session, participants will:

- - 1. Explain the approach to a variety of common pediatric presenting complaints
    2. Articulate an organized approach to and workup for the irritable infant
    3. Delineate several options to address procedural pain in the pediatric patient

1. **Approach to Shock**

By the end of the session, participants will:

- - 1. Understand the different physiologic causes and signs of shock
    2. Understand the difference between cardiogenic, neurogenic, hypovolemic and septic shock
    3. Learn the appropriate initial management for the different categories of shock

1. **Common Antibiotics and Their Use**

By the end of this session, participants will:

- - 1. Appreciate how the pharmacokinetics and pharmacodynamics of antibiotic therapy in sepsis are different than in other disease states
    2. Review and understand common pitfalls of commonly prescribed antibiotics in the emergency department

1. **Approach to Medical Education**

By the end of this session, participants will:

- - 1. Compare and contrast learning in residency with your prior educational experiences using principles from adult learning theory
    2. Discuss useful educational resources for resident learning in the current era
    3. Develop awareness of your role as teacher and teaching skills

1. **Approach to Chest Pain**

By the end of this session, participants will:

- - 1. Understand initial ED management of chest pain
    2. Understand high risk presentations of chest pain
    3. Practice basic ECG interpretation

1. **Endocrine Emergencies**

By the end of this session, participants will:

- - 1. Learn warning signs of most severe endocrine emergencies
    2. Understand initial ED management for common endocrine emergencies

1. **Giving Difficult News**

By the end of this session, participants will:

- - 1. Understand the difficulty in delivering bad news in the ED setting
    2. Practice breaking bad news in a mock patient encounter

1. **OB/GYN Emergencies**

By the end of this session, participants will:

- - 1. Understand common ED OB/GYN emergent presentations
    2. Learn ED management of common OB/GYN emergencies
    3. Understand radiologic imaging options and when to consult OB/GYN in the ED

1. **Neurologic Emergencies**

By the end of this session, participants will:

- - 1. Understand the intricacies of the ED neurologic exam
    2. Comprehend subtle neurologic findings
    3. Understand management of stroke, TIA and vertigo presentations

1. **Approach to Sepsis**

By the end of this session, participants will:

- - 1. Define sepsis
    2. Identify a source and start antibiotics
    3. Understand Vasopressor choices in sepsis

1. **Social Work – Understanding how and when to utilize your social worker**

By the end of this session, participants will:

- - 1. Never worry alone. Understand that if you are concerned about a patient’s wellbeing and feel that support from a social worker could help, just ask
    2. Understand how to talk to social work about the case
    3. Comprehend that everyone is a mandated-reporters. If you have worries or concerns about a patient’s safety, or potential abuse/neglect, please notify someone

1. **Evidence Based Medicine**

By the end of this session, participants will:

- - 1. Understand approach to reading clinical literature
    2. Understand how to apply evidence-based medicine to clinical practice

1. **Toxicology**

By the end of this session, participants will:

- - 1. Review how to recognize and treat toxidromes
    2. Review ECG changes associated with common overdoses and treatment options

1. **ENT Emergencies**

By the end of this session, participants will:

- - 1. Recognize true ENT emergencies
    2. Understand practical management of these emergencies
    3. Comprehend potential pitfalls and special circumstances (eg, anticoagulation in epistaxis) in the management of these conditions

1. **Ventilator Management**

By the end of this session, participants will:

- - 1. Understand the basic mechanism and controls of the ventilator
    2. Understand the various ventilation methods
    3. Know how to trouble shoot the ventilator

1. **Addiction Medicine**

By the end of this session, participants will:

- - 1. Understand the scope of addictions and risks for people with addictions in emergency departments
    2. Understand the disease model of addiction
    3. Understand a rationale for treating addictions in emergency settings and evidence-based strategies for doing so

1. **Dermatologic Emergencies**

By the end of this session, participants will:

- - 1. Recognize and treat common dermatological diagnoses in the emergency department
    2. Learn to recognize dermatological emergencies
    3. Learn common misdiagnoses in emergency medicine and primary care

1. **Understanding High Value Care**

By the end of this session, participants will:

- - 1. Understand concept of high value care in shared decision making
    2. Learn strategies to discuss high value care with patients

**Resident Led Didactics:**

1. **Consultant Etiquette**

By the end of this session, participants will:

- - 1. Understand how to efficiently and correctly present to a consultant
    2. Understand the need for a specific question when consulting
    3. Learn common errors and issues that arise with a difficult consult

1. **Common Pages and How to Respond**

By the end of this session, participants will:

- - 1. Learn what are typical pages an intern might receive both in the ED and on off-service rotations
    2. Understand which pages require immediate attention and response versus can be addressed after more urgent issues

1. **Resident Dilemmas and Resident Wellness**

By the end of this session, participants will:

- - 1. Understand the importance of wellness and strategies towards improving wellness during residency
    2. Discuss common dilemmas that arise in intern’s personal and professional lives

1. **Off-service Rotations: How to Succeed and Maximize Your Learning**

By the end of this session, participants will:

- - 1. Learn strategies towards maximizing the learning experience on each off-service rotation
    2. Understand what each particular service typically looks for in a rotating intern
    3. Learn how to best impress your future consultants

1. **Chief-Talk: Common Intern Problems and How to Avoid Them**

By the end of this session, participants will:

- - 1. Learn from the chief residents what are common intern mistakes in the beginning of residency
    2. Understand how to avoid being called into the Program Director’s office

**Skills Sessions:**

1. **Suturing**

Supplies required – plastic skin models, suturing kits and suture material

1. **Central Lines**

Supplies required – central line model with subclavian and internal jugular access, central venous catheter insertion kit, central venous catheter

1. **ECG workshop**

Supplies required – variety of different emergent ECGs

1. **Intubation**

Supplies required – intubation model, mac and miller blades, endotracheal tubes, bougie

1. **Lumbar puncture**

Supplies required – lumbar puncture model, lumbar puncture tray, spinal needle

1. **Pacing session**

Supplies required – external pacing box

1. **Splinting workshop**

Supplies required – plaster and fiberglass splinting material, ace wrap

1. **Thoracostomy**

Supplies required – chest tube insertion model, chest tube tray, scalpel, various chest tube sizes

**Simulation Cases:**

1. **40-year-old motor vehicle accident with polytrauma**

By the end of the session, participants will:

- - 1. Review ABCDE model of primary survey in ATLS
    2. Understand need for immediate intervention of tension pneumothorax
    3. Understand need for intubation for airway protection in altered mental status
    4. Correctly interpret FAST exam
    5. Understand role of immediate blood transfusion in hypotensive trauma patient with positive FAST

1. **55-year-old chest pain with inferior ST elevation myocardial infarction**

By the end of the session, participants will:

- - 1. Correctly interpret inferior STEMI ECG
    2. Review concepts of ACLS

1. **24-year-old asthma exacerbation**

By the end of the session, participants will:

- - 1. Understand respiratory support techniques in severe status asthmaticus
    2. Understand need for different ventilator settings in asthmatic patients

1. **16-year-old intentional tricyclic antidepressant overdose**

By the end of the session, participants will:

- - 1. Manage complex medical and social pediatric situation
    2. Understand management of TCA overdose
    3. Understand management of suicide attempt

**Ultrasound Curriculum:**

Topics covered:

1. **Trauma (FAST, e-FAST)**
2. **Abdominal aortic aneurysm (AAA)**
3. **Cardiac**
4. **Biliary**
5. **Deep vein thrombosis (DVT)**
6. **Procedural Guidance**
