# Supplementary material for: Impact of an end-of-fourth-year emergency medicine bootcamp
Source: Int J Emerg Med. 2021 Sep 3;14:48. doi: 10.1186/s12245-021-00371-8 (PMC8414734; doi:10.1186/s12245-021-00371-8)
Supplement: Supplementary file 2 — Additional file 2:. Weekly Bootcamp Schedule [file 12245_2021_371_MOESM2_ESM.docx]

Additional file 2 - Weekly Bootcamp Schedule

| **Monday** | **Tuesday** | **Wednesday** | **Thursday** | **Friday** |
| --- | --- | --- | --- | --- |
| Didactics  10a-12p  Ultrasound  1p-4p | Simulation  8a-12p  Didactics  1p-3p | Residency Program Didactics  8a-1p | Didactics  10a-12p  Procedural Skills Sessions  1p-3p | Didactics  10a-12p  Didactics  1p-3p |
