# Supplementary material for: Impact of an end-of-fourth-year emergency medicine bootcamp
Source: Int J Emerg Med. 2021 Sep 3;14:48. doi: 10.1186/s12245-021-00371-8 (PMC8414734; doi:10.1186/s12245-021-00371-8)
Supplement: Supplementary file 3 — Additional file 3:. End of Bootcamp Course Evaluation [file 12245_2021_371_MOESM3_ESM.docx]

Additional file 3 - End of Bootcamp Course Evaluation

**1. How has this course increased your confidence?**

Significantly

Moderately

Slightly

Not at all

**2. How has this course increased your competence?**

Significantly

Moderately

Slightly

Not at all

**3. How has this course impacted your procedural skills?**

Significantly

Moderately

Slightly

Not at all

**4. Would you recommend this course to next year's graduating class?**

Absolutely

Probably yes

Probably no

No

**5. Any other comments/suggestions**
