# Supplementary material for: Impact of an end-of-fourth-year emergency medicine bootcamp
Source: Int J Emerg Med. 2021 Sep 3;14:48. doi: 10.1186/s12245-021-00371-8 (PMC8414734; doi:10.1186/s12245-021-00371-8)
Supplement: Supplementary file 4 — Additional file 4:. Emergency Medicine Bootcamp New Intern Survey [file 12245_2021_371_MOESM4_ESM.docx]

Additional file 4 - Emergency Medicine Bootcamp New Intern Survey

**1. Did you participate in the EM bootcamp?**

Yes

No

**2. How do you feel your clinical knowledge compares to your co-interns?**

Significantly below

Moderately below

Average

Moderately above

Significantly above

**3. How do you feel your procedural skills compare to your co-interns?**

Significantly below

Moderately below

Average

Moderately above

Significantly above

**4. How confident were you at the start of internship?**

No confidence

Mild lack of confidence

Average

Mildly confident

Very confident
