# Supplementary material for: Impact of an end-of-fourth-year emergency medicine bootcamp
Source: Int J Emerg Med. 2021 Sep 3;14:48. doi: 10.1186/s12245-021-00371-8 (PMC8414734; doi:10.1186/s12245-021-00371-8)
Supplement: Supplementary file 5 — Additional file 5:. Emergency Medicine Bootcamp Program Director Survey [file 12245_2021_371_MOESM5_ESM.docx]

Additional file 5 - Emergency Medicine Bootcamp Program Director Survey

**1. Did your intern partake in EM bootcamp (specified in email sent to you)?**

Yes

No

**2. How does this intern's clinical knowledge compare to your other interns?**

Significantly below

Moderately below

Average

Moderately above

Significantly above

**3. How does this intern's procedural skills compare with your other interns?**

Significantly below

Moderately below

Average

Moderately above

Significantly above

**4. How would you rate this intern's confidence compared with other interns in your program?**

Significantly below

Moderately below

Average

Moderately above

Significantly above
